# A glimpse of the genetics of young‐onset Parkinson’s disease in Central Asia

**DOI:** 10.1002/mgg3.1671

**Published:** 2021-04-05

**Authors:** Rauan Kaiyrzhanov, Akbota Aitkulova, Jana Vandrovcova, David Murphy, Nazira Zharkinbekova, Chingiz Shashkin, Vadim Akhmetzhanov, Gulnaz Kaishibayeva, Altynay Karimova, Zhanybek Myrzayev, Malgorzata Murray, Talgat Khaibullin, John Hardy, Henry Houlden

**Affiliations:** ^1^ Department of Neuromuscular Disorders Institute of Neurology University College London London UK; ^2^ Department of Molecular Genetics National Center for Biotechnology Nur‐Sultan Kazakhstan; ^3^ Department of Clinical and Movement Neurosciences UCL Queen Square Institute of Neurology and The National Hospital for Neurology and Neurosurgery London UK; ^4^ Department of Neurology South Kazakhstan Medical Academy Shymkent Kazakhstan; ^5^ Contemporary Neurology and Neurorehabilitation Clinic "Shashkin Clinic" Almaty Kazakhstan; ^6^ Institute of Neurology and Neurorehabilitation Named After Smagul Kaishibayev Almaty Kazakhstan; ^7^ Kazakh Medical University of Continuing Study Almaty Kazakhstan; ^8^ Department of Neurology Semey Medical University Semey Kazakhstan

**Keywords:** age of onset, Central Asia, genetics, Kazakhstan, *LRRK2*, Parkinson's disease, Parkinson's disease genetics, young‐onset

## Abstract

**Background:**

Knowledge of the genetic background of many human diseases is currently lacking from genetically undiscovered regions, including Central Asia. Kazakhstan is the first Central Asian country where the genetic studies of Parkinson's disease (PD) have been emerging since it had become a member of the International Parkinson Disease Genomics Consortium. Here we report on the results of whole‐exome sequencing (WES) in 50 young‐onset PD (YOPD) cases from Kazakhstan.

**Methodology:**

WES was performed on 50 unrelated individuals with YOPD from Kazakhstan. Exome data were screened for novel/ultra‐rare deleterious variants in known and candidate PD genes. Copy number variants and small indels were also called.

**Results:**

Only three cases (6%) were found to be positive for known PD genes including two unrelated familial PD cases with *LRRK2* p.(Arg1441Cys) and one case with a homozygous pathogenic *PRKN* p.(Arg84Trp) variant. Four cases had novel and ultra‐rare variants of uncertain significance in *LRRK2*, *DNAJC13*, and *VPS35*. Novel deleterious variants were found in candidate Mendelian PD genes including *CSMD1*, *TNR*, *EIF4G1*, and *ATP13A3*. Eight cases harbored the East Asian‐specific *LRRK2* p.(Ala419Val) variant.

**Conclusions:**

The low diagnostic yield in our study might imply that a significant proportion of YOPD cases in Central Asia remains unresolved. Therefore, a better understanding of the genetic architecture of PD among populations of Central Asian ancestry and the pathogenicity of numerous rare variants should be further investigated. WES is a valuable technique for large‐scale YOPD genetic studies in Central Asia.

## Graphical abstract

Central Asia is an appealing region for PD genetic studies.

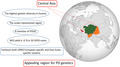

## 1 INTRODUCTION

The studies of genetic association with the disease have predominantly been performed in European populations. This European bias carries significant implications for disease risk predictions across global populations (Cavalli‐Sforza, 2005; Lu et al., 2014). The under‐representation of ethnically diverse populations precludes our ability to fully understand the genetic architecture of human disease and worsens health inequalities. This has been gaining particular importance in the field of Parkinson's disease (PD) genetics. International Parkinson Disease Genomics Consortium (IPDGC) is a research alliance focused on understanding the basis of PD and related disorders (Singleton, 2020). One of its multifaceted aims is to study diverse underrepresented ancestral groups. Kazakhstan, populated by a largely underrepresented, multinational Central Asian ancestral group, has recently become an IPDGC member through the collaboration with the Institute of Neurology University College London (IoN UCL). Currently, DNA samples from hundreds of PD cases and healthy controls from Kazakhstan are under genetic screening with the population‐customized NeuroChip genotyping array at IPDGC facilities in the United States. While the results of this screening will be available in due time, to get an unprecedented glimpse of the genetic background of Central Asian PD, here we report the findings from whole‐exome sequencing (WES) in young‐onset PD (YOPD) from Kazakhstan.

## 2 MATERIALS AND METHODS

DNA samples of 50 unrelated individuals with YOPD were retrieved from the research‐ready genetic database of Kazakhstani PD cases stored at IoN UCL, the main collaborator of Kazakhstan on the genetic studies of neurodegenerative disorders. EOPD was defined as the onset before the age of 50 years (Schrag & Schott, 2006). WES was performed at IoN UCL and variants were annotated and filtered as previously described by Li et al., 2020. Copy number variants (CNVs) were called in *SNCA* (*163890), *PRKN* (*602544), *PINK1* (*608309), *ATP13A2* (*610513), *DJ1* (*602533), *TH* (*191290), *VPS35* (*601501), *GRN* (*138945), and *HMOX1* (*141250) genes and small indels were called in 13 PD genes (Supplementary for Methods). Parental DNAs of the affected individuals included in this study were not available. Exome data were also screened for previously reported candidate Mendelian PD genes (Chew et al., 2019; Farlow et al., 2016; Jansen et al., 2017; Kun‐Rodrigues et al., 2015; Quadri et al., 2014; Sandor et al., 2017; Schormair et al., 2018; Siitonen et al., 2017; Trinh et al., 2019; Yemni et al., 2019). Positive variants were checked in 350 WES data of Central Asian control subjects. This study was approved by the Research Ethics Committees of the National Center for Biotechnology (NCB) Kazakhstan (4/29.08.2017) and the Institute of Neurology University College London (IoN UCL) (07/Q0512/26).

## 3 RESULTS

The mean age at PD onset in the cohort was 38.1 ± 7.5 years (range 14–50), the mean age at examination was 46.4 ± 7.7, and the mean disease duration was 8.3 ± 4.7 (Table 1). The cohort was made of 38 Kazakhs, 11 ethnic Russians, and 1 ethnic Korean. Family history of PD was present in 10 (21%) cases.

**TABLE 1:** Clinico‐demographic characteristics of the cohort

Demographic and clinical characteristics	Values
Mean age at onset ±SD(years), (range)	38.1 ± 7.5 (14–50)
Mean age at the last examination ±SD(years), (range)	46.4 ± 7.7 (28–66)
Mean disease duration ±SD(years), (range)	8.3 ± 4.7 (0–24)
Male to female ratio	1:0.8 (26 M:22F)
Family history, n (%)	10 (21)
Mean Hoehn‐Yahr stage	2.4 ± 0.6
Mean motor MDS UPDRS score	40.1 ± 18.3

Abbreviaitons: F, females; M, males; MDS UPDRS, Movement disorders society unified Parkinson's disease rating scale; n, number; SD, standard deviation.

Only three cases were found to harbor pathogenic variants in known PD genes (Table 2). *LRRK2* (*609007) (NM_198578.4) c.4321C>T, p.(Arg1441Cys) variant was found in two unrelated Kazakhs with familial PD and age of onset at 39 years. One ethnic Russian case had a homozygous pathogenic missense variant in *PRKN* (NM_004562.3) c.250C>T, p.(Arg84Trp). This variant is predicted to result in nonsense‐mediated decay and was absent in 19916 of East Asian alleles in gnomAD.

**TABLE 2:** Variants in known PD genes

Gene	*LRRK2*	*PRKN*	*LRRK2*	*LRRK2*	*LRRK2*	*DNAJC13*	*VPS35*
Transcript ID	NM_198578.4	ENST00000338468.7	NM_198578.4	NM_198578.4	NM_198578.4	NM_015268.4	NM_018206.6
Variant	c.4321C>T; p.(Arg1441Cys)	c.250C>T; p.(Arg84Trp)	c.1256C>T; p.(Ala419Val)	c.4001G>A; p.(Arg1334Gln)	c.3812C>T; p.(Thr1271Ile)	c.6211C>T; p.(Arg2071Trp)	c.71C>G; p.(Pro24Arg)
Zygosity of the variant	Het	Hom	Het	Het	Het	Het	Het
Number of positive cases	2	1	8	1	1	1	1
Family history	Yes (2)	No	No (8)	No	No	No	No
Age at onset (y.o.)	39 (2)	38	Mean 42.6	49	43	40	35
Ethnic group	Kazakhs (2)	Russian	Kazakhs (3), Russians (4), Russian‐Kazakh (1)	Kazakh	Kazakh	Kazakh	Korean
gnomAD allele frequency	0.00001 (Het allele count – 1)	0.001972 (Het allele count – 557), absent in 19916 alleles from East Asia	0.00048 (Het allele count – 137)	0.000014 (Het allele count −4)	absent	0.0000079 (Het allele count‐ 2)	Absent
rs number	rs33939927	rs34424986	rs34594498	rs772964685	absent	rs377206231	Absent
Clin significance (accessed: 05.03.2021)	Pathogenic	Pathogenic	Benign	Not reported	Not reported	Not reported	Not reported
Varsome (accessed: 05.03.2021)	Likely Pathogenic	Pathogenic	Likely Benign	Uncertain significance	Uncertain significance	Uncertain significance	Uncertain significance
Sift	0.07	Nonsense‐mediated_decay; Significant alteration of ESE / ESS motifs ratio (−9)	0.02	0	0	0.02	Nonsense_mediated_decay, Activation of a cryptic Donor site. Potential alteration of splicing
Polyphen	0.997		0.492	0.998	0.993	0.859	
CADD phred	22.3		24.1	32	25	26.3	
GERP++RS	4.74		5.12	5.56	5.82	5.98	
FATHMM score	−1.38		1.34	1.46	2.87	0.81	
LRT score	0.000686		0.000085	0	0	0	
MetaLR score	0.6287		0.0827	0.6487	0.0781	0.2351	
MutPred score	—		—	0.447	0.353	—	
Mutation tester score	0.999504		0.813121	1	1	0.999997	
PROVEAN score	−3.23		−1.32	−2.53	−3.48	−1.74	
REVEL score	0.66		0.175	0.669	0.227	0.242	

Abbreviations: Het, heterozygous; Hom, homozygous; y.o., years old.

Four cases had variants of uncertain significance (VUS) in *LRRK2*, *DNAJC13* (*614334), and VPS35 (*601501). This included two novel and ultra‐rare (gnomAD allele frequency 0.00001) deleterious *LRRK2* variants: c.3812C>T, p.(Thr1271Ile) and c.4001G>A, p.(Arg1334Gln) found in two Kazakh PD cases. While p.(Thr1271Ile) is located in the leucine‐rich repeat domain, p.(Arg1334Gln) resides in the Ras of complex protein domain of the LRRK2 protein. Ultra‐rare and novel deleterious variants in *DNAJC13* (NM_015268.4) c.6211C>T, p.(Arg2071Trp) and VPS35 (NM_018206.6) c.71C>G, p.(Pro24Arg) were found in other two cases in (Table 2).

Novel deleterious variants were found in candidate Mendelian PD genes including *CSMD1* (*608397), *TNR* (*601995), *EIF4G1* (*600495) (one of the variants was found by calling small indels), and *ATP13A3* (*610232) (Table S1). Additionally, rare and novel deleterious missense and loss‐of‐function variants were found in genes linked to pathways involved in PD (Table S2). None of the rare and novel variants found in this study were present in 350 Central Asian control subjects.

Eight affected individuals carried *LRRK2* c.1256C>T, p.(Ala419Val), the variant that has previously been thought to increase PD risk in East Asians. This variant was found in four Russians, three Kazakhs, and one case of mixed Kazakh‐Russian ethnic origin. They did not have a family history of PD and the mean age of PD onset for them was 42.6 years old. A common genetic susceptibility variant in Asian PD cohorts, *LRRK2* c.7153G>A, p.(Gly2385Arg), was not found in the present study. Calling CNVs in nine PD‐associated genes did not reveal any positive cases.

## 4 DISCUSSION

A number of human migration waves, spanning from the prehistoric era to early modern times, have commenced, terminated, or passed through Central Asia, leaving a significant mark on the genetics of the region (Martínez‐Cruz et al., 2011). Genetic diversity in Central Asia is among the highest in Eurasia making this region appealing for launching genetic studies including PD genetics (Comas et al., 2004). Kazakhstan serves as a gateway to Central Asia as it is the first country in the region where PD genetics has been emerging.

WES has evolved into a suitable approach for the study of both rare monogenic and common sporadic forms of PD. Several studies performed WES in YOPD cases and the diagnostic yield ranged between 3.5% and 44% when considering pathogenic, likely pathogenic variants, high‐impact risk factor variants, and VUS in known Mendelian PD genes (Gustavsson et al., 2017; Li et al., 2020; Quadri et al., 2014; Sandor et al., 2017; Schormair et al., 2018; Trinh et al., 2019).

In our study, the diagnostic yield was 6% when focusing solely on pathogenic variants in known Mendelian PD genes and this number was close to the findings of the recent WES study in the East Asian YOPD cohort (Li et al., 2020). Eight percent of cases in the present report had rare VUS in the established PD genes. There could be several reasons for the low diagnostic yield in our study. Firstly, pathogenic variants might reside in intronic or intragenic regions escaping the coverage of exome sequencing. Secondly, repeat polymorphisms, structural variants, or the true pathogenic variants might not be in currently known PD‐causing genes.

While pathogenic variants in autosomal‐recessive PD genes such *PRKN*, *PINK1*, *DJ1* are relatively rare in the general PD population, they have been reported to be responsible for a substantial proportion of YOPD worldwide. Thus, *PRKN* accounts for 8.6% of YOPD cases, followed by *PINK1* (3.7%) and DJ1 (0.4%). Furthermore, both *PRKN* and *PINK1*, and to the lesser extent *DJ1*, appear to be commonly implicated in Asian YOPD cases (Cherian & Divya, 2020; Lim et al., 2019). In our report, only one case of the self‐reported Russian ethnicity was found to harbor a homozygous pathogenic variant in *PRKN*. Exome sequencing along with calling CNVs and small indels did not reveal any other cases with known AR PD genes. The absence of AR PD genes among Kazakhs, comprising 76% of the current cohort, might be explained by the extremely low rates of consanguinity in Kazakhs compared to the rest of the Central Asian populations (Saira et al., 2015). The low diagnostic yield in our study might imply that a significant proportion of YOPD cases in Central Asia remains unresolved. Therefore, a better understanding of the genetic architecture of PD among populations of Central Asian ancestry and the pathogenicity of numerous rare variants should be further investigated.

The frequent *LRRK2* p.(Arg1441Cys) variant, as well as *LRRK2* p.(Ala419Val) variant, were present in Kazakhstani YOPD cases (4% and 16% of the cohort, respectively). Considering that *LRRK2* p.(Arg1441Cys) has been infrequent in East Asian PD populations and *LRRK2* p.(Ala419Val) has rarely been reported in European and West Asian PD cohorts (Lim et al., 2020; Paisán‐Ruiz, 2009; Peng et al., 2017; Shu et al., 2019; Tan et al., 2006), the presence of both variants in a small sample of Kazakhstani PD cases highlights the unique genetic background of Central Asia populations. Similar to East Asian PD populations, the present cohort was negative for *LRRK2* c.6055G>A, p.(Gly2019Ser).

*LRRK2* p.(Ala419Val) has initially been reported as a pathogenic variant conferring risk of YOPD in East‐Asians (Li et al., 2015). Although, the contribution of this variant to PD risk in Asians has been a contentious subject with some East Asian studies considering this variant as a putatively nonpathogenic population‐specific single nucleotide polymorphism (Kaiyrzhanov et al., 2020). Recently, this variant was reclassified as benign in ClinVar (https://www.ncbi.nlm.nih.gov/clinvar/variation/39131/). Interestingly, the minor allele frequency for *LRRK2* p.(Ala419Val) was suggested to be higher in Central Asia compared to East Asian populations (0.02 in PD cases and 0.012 in healthy controls for Central Asia vs 0.008 in PD cases and 0.004 in healthy controls for East Asia). Additionally, the allelic and genotypic frequencies for *LRRK2* p.(Ala419Val) did not statistically differ between PD cases and controls in Kazakhstan (odds ratio – 1.5, 95% CI: 0.4941–4.5463, *p*=0.4), suggesting that this variant is less likely to be a genetic susceptibility variant for PD in Central Asia (Kaiyrzhanov et al., 2020).

WES has led to the identification of more than 40 candidate genes in PD, though only three of them are currently supported with functional evidence (*VPS35*, *TMEM230*, and *DNAJC13*) (Yemni et al., 2019). Identifying rare predicted‐damaging variants in the reported candidate Mendelian PD genes in the Central Asian YOPD cohort might provide further supporting evidence for their disease‐causing role in PD across different populations. Although segregation analysis is required in our study to validated these variants.

The diagnostic yield of WES studies in YOPD has generally been low, suggesting that a large number of early‐onset cases still remain to be explained. Despite the possibility of overlooking some variants due to the limitations of WES technology, it is a preferable approach in the study of YOPD genetics and its diagnostic yield could be increased by regular inspection for novel gene‐disease associations emerging in the literature (Need et al., 2017).

In conclusion, here we report on the results of WES in a sample of YOPD cases from Central Asia and provide the rationale for further large‐scale YOPD genetics studies in the region.

## CONFLICT OF INTERESTS

The authors have declared no conflict of interest.

## AUTHOR CONTRIBUTIONS

RK collected cases, analyzed WES data, wrote the manuscript. AA extracted DNAs from the blood samples of the cases, contributed to the tables. JV, DM made a bioinformatic analysis of raw WES data, called CNVs and small indels. NZ, CS, VA, GS, AK, ZM, TK, MM recruited patients and provided clinical information and material. JH, HH designed the study and interpreted the data, and provided a critical review of the manuscript.

## Supporting information

Table S1‐S2

Supplementary Material

## Data Availability

The data that support the findings of this study are available from the corresponding author, upon reasonable request.
