# Supplementary material for: A glimpse of the genetics of young‐onset Parkinson’s disease in Central Asia
Source: Mol Genet Genomic Med. 2021 Apr 5;9(6):e1671. doi: 10.1002/mgg3.1671 (PMC8222829; doi:10.1002/mgg3.1671)
Supplement: Supplementary file 1 — Table S1‐S2 [file MGG3-9-e1671-s001.docx]

**Supplementary Table 1**. Variants in candidate Mendelian PD genes

| **Gene** | *CSMD1* | *TNR* | *EIF4G1* | *EIF4G1* | *EIF4G1* | *ATP13A3* |
| --- | --- | --- | --- | --- | --- | --- |
| **Transcript ID** | NM_033225.6 | NM_003285.3 | NM_182917.4 | NM_001194946.2 | ENST00000342981 | NM_024524.3 |
| **Variant** | c.17G>T; p.(Gly7Trp) | c.185C>T; p.(Pro62Leu) | c.126_137del; p.(Asn42_Gln46delinsLys) | c.3481C>T; p.(Arg1161Cys) | c.126_137del; p.(Asn42_Gln46delinsLys) | c.3079del; p.(Trp1027GlyfsTer31) |
| **Zygosity of the variant** | Het | Het | Het | Het | Het | Het |
| **Zygosity of previously reported PD-associated variants** | De novo | Het | Het | Het | Het | Het |
| **Resource** | PMID: 28808687 | PMID: 26595808 | PMID: 25765080 | PMID: 25765080 | PMID: 25765080 | PMID: 29505581 |
| **gnomAD allele frequency** | absent | absent | absent | 0.000007 | absent | absent |
| **Queen Square Genomics database** | absent | absent | absent | absent | absent | absent |
| **rs number** | absent | absent | absent | rs1425712161 | absent | rs76830047 |
| **Sift** | 0.03 | 0 | - | 0 | indel | - |
| **Polyphen** | 0.998 | 0.999 | - | 0.893 |  | - |
| **CADD phred** | 24.7 | 32 | - | 26.3 |  | - |
| **GERP++RS** | 5.26 | 5.28 | - | 3.4 |  | - |
| **FATHMM score** | 1.67 | 1.46 | - | 3.83 |  | - |
| **LRT score** | 0.000001 | 0 | - | 0.000023 |  | - |
| **MetaLR score** | 0.1911 | 0.2046 | - | 0.0374 |  | - |
| **MutPred score** | - | 0.484 | - | 0.377 |  | - |
| **Mutation tester score** | 0.999 | 1, 1 | - | 1 |  | - |
| **PROVEAN score** | -3.68 | -5.94 | - | -3.04 |  | - |
| **REVEL score** | 0.262 | 0.551 | - | 0.178 |  | - |

**Supplementary Table 2**. Variants in genes linked to pathways involved in PD

| **Gene** | *COL24A1* | *SPAG17* | *PAK4* | *TRPM2* | *NCKIPSD* | *PLEKHM1* |
| --- | --- | --- | --- | --- | --- | --- |
| **Transcript ID** | NM_152890.7 | NM_206996.4 | NM_001014831.2 | NM_001320351.1 | NM_016453.4 | XP_016880941.1 |
| **Variant** | c.649G>C; p.(Gly217Arg) | c.5804C>T; p.(Pro1935Leu) | c.170C>T; p.(Ala57Val) | c.1303C>T;  p.(Gln435Ter) | c.545C>T; p.(Pro182Leu) | c.82C>T;  p.(Arg28Cys) |
| **Zygosity** | Het | Het | Het | Het | Het | Het |
| **Resource** | PMID: 30256453 | PMID: 27402877 | PMID: 27903866 | DOI https://doi.org/10.9734/AIR/2014/10543, PMID: 30037128, PMID: 27957685 | PMID: 28892059 | PMID: 24057672, PMID: 25498145,PMID: 25574946, PMID: 25061481 |
| **gnomAD allele frequency** | Het allele count - 1 | Het allele count - 1 | Absent |  | Het allele count - 1 | Het allele count - 2 |
| **rs number** | rs752077479 | rs773871021 | rs963991135 | - | rs775305129 | rs370132107 |
| **Sift** | 0 | 0 | 0.05 | - | 0.01 | 0 |
| **Polyphen** | 1 | 0.998 | 0.463 | - | 0.997 | 1 |
| **CADD phred** | 25.6 | 24.1 | 21.2 | 42 | 23.9 | 28 |
| **GERP++RS** | 5.61 | 4.9 | 1.56 | 4.14 | 4.17 | 5.03 |
| **FATHMM score** | -4.07 | 1.82 | -2.06 | - | -0.53 | 2.35 |
| **LRT score** | 0.001555 | 0.003145 | 0.076223 | 0.000003 | 0.00011 | 0 |
| **MetaLR score** | 0.9304 | 0.1177 | 0.0621 | - | 0.5864 | 0.1535 |
| **MutPred score** | 0.76 | - | 0.541 | - | 0.428 |  |
| **Mutation tester score** | 1, 1 | 0.999921 | 0.994808 | 1, 1, 1, 1 | 1, 1, 1 | 0.9999 |
| **PROVEAN score** | -5.83 | -4.73 | -2.23 | - | -8.45 | -1.59 |
| **REVEL score** | 0.912 | 0.175 | 0.054 | - | 0.444 | 0.433 |

**Supplementary Table 2**. Variants in genes linked to pathways involved in PD (continued)

| **Gene** | *ATP5A1* | *PIK3CG* | *ADAMTS19* | *EIF2A* | *SEC31A* | *PHOX2B* |
| --- | --- | --- | --- | --- | --- | --- |
| **Transcript ID** | NM_007505 | XP_016867817.1 | NM_133638 | ENST00000490505.1 | NM_001318119 | [NM_003924.4](http://www.ncbi.nlm.nih.gov/nuccore/NM_003924.4) |
| **Variant** | c.80A>C; p.(His27Pro) | c.532C>T; p.(Arg178Cys) | c.1311-3_1311-1del | c.149G>A; p.(Gly50Asp) | c.2633A>T; p.(Ter878LeuextTer12) | c.768_773dup;  p.(Ala259_Ala260dup) |
| **Zygosity** | Het | Het | Het | Het | Het | Het |
| **Resource** | PMID: 28916538 | PMID: 28802037 | PMID: 27845893 |  | PMID: 31316342 | PMID: 16021468 |
| **gnomAD allele frequency** | Absent | Het allele count - 1 | Absent | Absent | Absent | Absent |
| **rs number** | - | rs1157393554 | - | - | - | - |
| **Sift** | - | 0 | - | Nonsense mediated decay | - | - |
| **Polyphen** | 0 | 0.991 | - |  | - | - |
| **CADD phred** | - | 29.4 | - |  | 14.01 | - |
| **GERP++RS** | - | 5.33 | - |  | 4.31 | - |
| **FATHMM score** | - | -1.01 | - |  | - | - |
| **LRT score** | - | 0 | - |  | - | - |
| **MetaLR score** | - | 0.6254 | - |  | - | - |
| **MutPred score** | - | 0.596 | - |  | - | - |
| **Mutation tester score** | - | 1, 1, 1 | - |  | 1, 1, 1 | - |
| **PROVEAN score** | - | -5.33 | - |  | - | - |
| **REVEL score** | - | 0.593 | - |  | - | - |
