# Supplementary material for: A glimpse of the genetics of young‐onset Parkinson’s disease in Central Asia
Source: Mol Genet Genomic Med. 2021 Apr 5;9(6):e1671. doi: 10.1002/mgg3.1671 (PMC8222829; doi:10.1002/mgg3.1671)
Supplement: Supplementary file 2 — Supplementary Material [file MGG3-9-e1671-s002.docx]

**Supplementary methods**

**CNV calling methods**

The CNV analysis was performed using a combination of two established methods for CNV detection CoNIFER(Krumm N, Sudmant PH, Ko A, O'Roak BJ, Malig M, Coe BP, NHLBI Exome Sequencing Project, Quinlan AR, Nickerson DA and Eichler EE, 2012. Copy number variation detection and genotyping from exome sequence data. Genome Research, doi:10.1101/gr.138115.112) and ExomeDepth (Plagnol V, Curtis J, Epstein M, Mok K, Stebbings E, Grigoriadou S, Wood NW, Hambleton S, Burns SO, Thrasher A, Kumararatne D, Doffinger R, Nejentsev S: A robust model for read count data in exome sequencing experiments and implications for copy number variant calling. Bioinformatics. 2012, 28: 2747-2754) using default settings. CoNIFER implements singular value decomposition while ExomeDepth identifies CNVs using read depth data and relies on a reference dataset for normalisation. Unrelated samples without a PD diagnosis that were processed using the same library capture and sequenced in the same batch were used as controls. All CNVs were extracted from bam files generated as described above but only variants overlapping known PD causing genes which were further prioritised. All variants were annotated using gnomAD – SV (Collins, R.L., Brand, H., Karczewski, K.J. et al. A structural variation reference for medical and population genetics. Nature 581, 444–451 (2020). <https://doi.org/10.1038/s41586-020-2287-8>).

**Small Indel calling methods**

Paired-end reads are aligned to the GRCh38 human reference using the Burrows-Wheeler Aligner (BWA-MEM https://doi.org/10.1093/bioinformatics/btp324 ) and processed using the GATK best-practices to create BAM files. https://www.ncbi.nlm.nih.gov/pmc/articles/PMC4243306/

Variants were separately called using GATK 4.1.4.0 and Freebayes-v1.3.1 ( https://arxiv.org/abs/1207.3907 ) for padded intervals based on known PD causing genes.

For GATK variants were called using joint variant calling from gvcf files. For Freebayes variants were called for each sample individually directly from the source bam file.
